# Capturing Structure and Morphology in Responsive Microgels: From Intrinsic Free Energy to Collective Behavior

**DOI:** 10.1021/acs.macromol.5c01988

**Published:** 2025-10-06

**Authors:** Arturo Moncho-Jordá, Alejandro Cuetos, Miguel A. Fernandez-Rodriguez, Joachim Dzubiella, Alessandro Patti

**Affiliations:** † Department of Applied Physics, 16741 Universidad de Granada, Campus Fuentenueva S/N, 18071 Granada, Spain; ‡ Institute Carlos I for Theoretical and Computational Physics, Universidad de Granada, Campus Fuentenueva S/N, 18071 Granada, Spain; § Center for Nanoscience and Sustainable Technologies (CNATS), and Department of Physical, Chemical and Natural Systems, 16772 Universidad Pablo de Olavide, 41013 Sevilla, Spain; ∥ 191253 Physikalisches Institut, Albert-Ludwigs-Universität Freiburg, Hermann-Herder Straße 3, D-79104 Freiburg, Germany; ⊥ Department of Chemical Engineering, The University of Manchester, Manchester M13 9PL, U.K.

## Abstract

We develop a coarse-grained theoretical and computational framework based on responsive effective pair potentials to describe the compression behavior of core–shell microgels in a good solvent. Our approach accounts for the intrinsic morphological heterogeneity of the particles by decomposing the total free energy into core and shell contributions, each governed by a Flory–Rehner-type model with distinct mechanical properties. Mechanical equilibrium between both regions is imposed to capture the swelling behavior self-consistently. Interparticle interactions are modeled using a four-component, size-dependent multi-Hertzian pair potential that incorporates the differential mechanical response and compressibility of the core and shell. The model parameters are determined by fitting to dynamic light scattering measurements of PNIPAM microgels across a range of temperatures spanning the lower critical solution temperature, thus, capturing the thermoresponsive swelling behavior. The model also provides variation of the core size upon thermal or mechanical collapse of the microgels. Monte Carlo simulations are then performed to investigate the collective properties of concentrated suspensions, including size distribution, effective packing fraction, structural organization, and phase behavior as a function of compression. Our results demonstrate that both the intrinsic particle softness and responsiveness, as well as their heterogeneous internal structure, play a crucial role in determining the microstructure and thermodynamic state of dense microgel systems.

## Graphical abstract

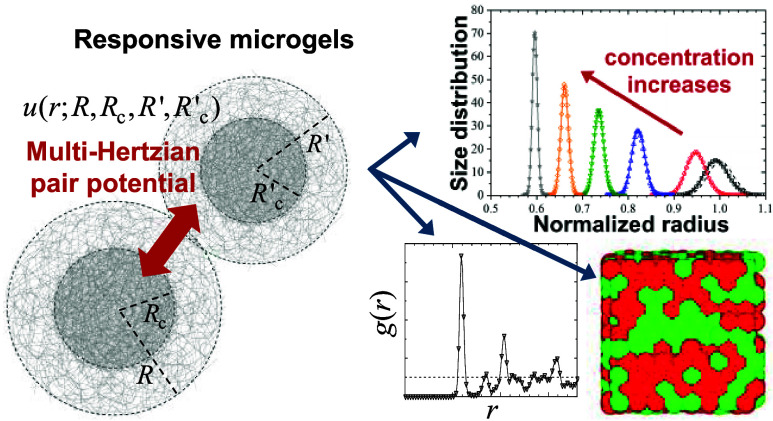

## Introduction

Suspensions of responsive colloids have attracted considerable interest in the field of soft-matter science due to the ability to adapt to changes in the environmental conditions.^1^ Responsiveness implies that particles possess one or more internal degrees of freedom (DoF) that allow for significant alterations in their internal and collective dynamical properties of the system.^2–16^ These DoFs encompass various features including the particle’s conformation,^17–20^ size,^21–25^ shape,^26–32^ orientation,^33,34^ charge density,^23,35^ or electric dipole moment,^5^ among others. They are directly influenced by interactions with other particles or external fields, resulting in colloids capable of undergoing significant changes in their internal distributions.

Microgel suspensions, consisting of particles formed by cross-linked polymer chains, are among the most emblematic soft-matter systems, characterized by the responsive behavior of their individual particles. In this case, the particle size, represented by hydrodynamic radius (*R*), stands out as the main relevant internal DoF.^23,36–42^ It is widely recognized that even minor alterations in temperature or pH can trigger a swollen-to-deswollen transition, resulting in a significant change in the particle radius, often by a factor of at least two from the collapsed to the swollen state. Microgel radius can also be tuned by increasing particle concentration and/or the particle softness, leading to a progressive reduction induced by the excluded volume repulsions with the surrounding particles, with relevant implications on the structural and dynamical properties of the suspension.^14–16,22,23,32,41–49^ In addition, changes in particle size are of great importance in various applications, such as those involving permeable microgels to control the release kinetics in drug delivery^50–54^ and hydrogel-based colloidal nano- or microreactors to tune the reaction rate in selective catalysis.^55–57^

In particular, poly­(*N*-isopropylacrylamide) (PNIPAM) based microgel suspensions are one of the most extensively studied, mainly because these particles exhibit a volume transition in water from a swollen to collapsed state when the temperature is increased above the so-called lower critical solution temperature (LCST), close to the human body temperature (of about 32 °C).^58,59^ When the temperature decreases below *T*_LCST_, the PNIPAM chains become hydrated, causing the microgel particle to swell as it absorbs a large amount of water. Conversely, if the temperature rises above this threshold, then molecular agitation disrupts the hydrogen bonding between water and the amide groups. This breakdown destabilizes the water structure around the PNIPAM chains, inducing hydrophobic interactions between the isopropyl groups. As a result, the polymer network collapses and expels the water it contains.^60^ Given their versatility, PNIPAM microgels have served as fundamental model systems, paving the way for numerous advancements in the field of soft responsive materials.^61^

The fact that microgel particles are responsive colloids also implies that the particle size is controlled by an intrinsic free energy, *F*(*R*), representing the energy cost involved in the swelling/deswelling of a single isolated colloid.^22,24,25^ Usually, *F*(*R*) shows a minimum located at a certain reference state with size *R*_eq_, representing the particle size in equilibrium of an infinite diluted suspension.^37,62^ The *F*(*R*) from Flory–Rehner theory for homogeneous microgels interacting with a Hertzian potential was implemented by Urich and Denton in pioneering Monte Carlo simulations with explicit resolution of *R* to study swelling, structure, and phase stability of compressible microgels.^22^ Related studies have used Brownian dynamics simulations.^25^ This free energy landscape permits thermal fluctuations of the particle size around *R*_eq_, resulting in a continuous polydisperse size distribution, which we refer to as the parent size distribution. It is given by

1 $$p\left(R\right)=qe^{-\beta F\left(R\right)}$$

where *q* is a constant prefactor (units of length^–1^) to fulfill normalization, i.e., ∫*p*(*R*)­d*R* = 1, and β = 1/(*k*_*B*_ *T*) (*k*_*B*_ is the Boltzmann constant and *T* the absolute temperature). Therefore, suspensions of microgel particles are inherently polydisperse, even when the individual particles are identical. The shape of *F*(*R*) around *R*_eq_ determines the softness of the microgel particles. If *F*(*R*) exhibits a pronounced, sharp curvature near *R*_eq_, this indicates that the microgel behaves as a very stiff colloid, making it uncommon to find particles with sizes significantly different from those of *R*_eq_. This state can be experimentally achieved by increasing the cross-linker concentration within the polymer network of the microgel or by inducing the particle’s collapse at *T* < *T*_LCST_. In contrast, if *F*(*R*) shows a broader profile, then the microgel exhibits a softer behavior, allowing for significant size fluctuations. In this case, the particles are more likely to deform, for example, by increasing the particle concentration.^40,63^

We emphasize that *p*(*R*) represents the probability size distribution of a suspension of microgels in the limit of infinite dilution so that microgel-microgel interactions are negligible. However, for more dense suspensions, these pair interactions will also affect the particle size, inducing the compression of the particles. We will denote *f*(*R*) as the actual size probability distribution of the system at this particle concentration.

One of the most important morphological features of microgels is their core–shell internal structure. In this work, we use the terms core and shell to designate the internal and external domains of the particle, respectively, distinguished by a significant disparity in polymer volume fractions (see Table 1 for details). Typically, microgels exhibit a denser, highly cross-linked core with nearly uniform polymer density, surrounded by a loosely cross-linked shell where the density gradually decreases to zero. These two regions differ not only in polymer density but also in the number and length of polymer chains, resulting in markedly different mechanical properties. For example, the Young’s modulus­(which governs the stiffness of repulsive interactions) and the bulk modulus which determines the internal compressibility of the polymer network) are significantly higher in the core than in the shell.

**Table 1:** Key Parameters of the Theoretical Model Used in the Computer Simulations Along with the Values Obtained from Fitting to Experimental Data^a^

parameter	symbol	value
relation between monomer and solvent volume	*B*	3.644
fitting parameters of χ (eq 41)	χ_1_, χ_0_, *κ*, *T*_χ_	0.17, 0.68, 0.242 °C^–1^, 26.9 °C
equilibrium radius of the particles in the swollen state	*R* ^sw^	345 nm
equilibrium radius of core the particles in the swollen state	*R*_c_^sw^	207 nm
number of chains in the shell region	*N*_ch,s_	26.2
number of chains in the core region	*N*_ch,c_	120.4
packing fraction of the core in the collapse state	ϕ_0c_	0.625
packing fraction of the shell in the collapse state	ϕ_0s_	0.271
packing fraction of the core in the swollen state	ϕ_c_^sw^	0.153
packing fraction of the shell in the swollen state	ϕ_s_^sw^	0.044
fraction of charged monomers in the core region	*f*_c_	0.0
fraction of charged monomers in the shell region	*f*_s_	0.601
average number of monomers per chain in the core region	*n*_c_	5.35
average number of monomers per chain in the shell region	*n*_s_	25.59
lower critical solution temperature for PNIPAM	*T*_LCST_	30.57 °C
radius of the particles in the collapse state	*R*_0_	200 nm
radius of core in the collapse state	*R*_0*c*_	129 nm
proportional constant to adjust the theoretical model	*C*	30
fitting parameters of *R*_c_ as a function of *R* (eq 42)	*A*_1_, *A*_2_, *A*_3_, *A*_4_	0.604, 1.05, 0.712, 4.06

a See main text for further details.

In recent years, the compression behavior of core–shell microgels in good solvents has been extensively studied through coarse-grained computer simulations using effective pair potentials. Several models incorporate particle responsiveness, enabling size reduction under compression, and have significantly contributed to our understanding of microgel behavior in dense suspensions.^22,32,41,64^ However, many of these approaches simplify the internal structure of the microgel by not explicitly including the core–shell morphology in the formulation of the intrinsic free energy landscape. Moreover, interparticle interactions are often modeled using an effective repulsive Hertzian potential.^65^ While the Hertzian model provides an accurate description of the repulsion between homogeneous elastic spheres, it does not fully capture the structural heterogeneity and mechanical anisotropy inherent in real microgels. Alternative approaches have introduced multi-Hertzian pair potentials to differentiate the mechanical responses of the core and shell regions.^49,66^ These models offer a more refined description of internal structure but are generally nonresponsive, meaning that the interaction potentials do not adapt to particle compression.

In this work, we build upon these earlier contributions and propose a coarse-grained model based on effective pair potentials that incorporate both the core–shell morphology and the responsive nature of microgels under good solvent conditions. Our approach adopts a modified Flory–Rehner free energy framework,^41,62^ in which the total intrinsic free energy *F*(*R*) is expressed as the sum of two contributions corresponding to the core and the shell. Mechanical equilibrium between the two regions is enforced to ensure thermodynamic consistency. Furthermore, we show that an accurate description of concentrated microgel suspensions requires a four-component size-dependent multi-Hertzian pair potential that reflects the distinct elastic responses and responsiveness of the core and shell domains.

After developing the theoretical model that couples intrinsic swelling and interparticle interactions, we determined the relevant physical parameters by fitting to experimental swelling data of dilute PNIPAM microgel suspensions across a range of temperatures (below and above the LCST), obtained via dynamic light scattering (DLS). To the best of our knowledge, this is the first application of a free energy model that explicitly accounts for the distinct swelling behaviors of the core and shell to describe the thermal collapse of microgels. We also impose mechanical equilibrium conditions inside the microgel to characterize the collapse of a swollen microgel induced by external pressure, determining how the radius of the microgel core shrinks upon compression. Finally, we conduct Monte Carlo simulations that incorporate particle responsiveness to analyze how compression influences the size distribution, effective packing fraction, microstructure, and phase behavior of core–shell microgels in concentrated suspensions under good solvent conditions.

## Theory

### Model for the Polymer Distribution of a Swollen Microgel

In this section, we introduce a simplified model to describe the polymer density profile within a microgel immersed in a good solvent, for which the particle is in the swollen state. We assume that the microgel is isolated; i.e., it does not interact with other particles in the suspension (dilute regime). The effect of interparticle interactions will be incorporated in subsequent sections.

Microgels are composed of a densely cross-linked, uniform core surrounded by a polymeric shell, where the polymer concentration gradually decreases to zero with increasing radial distance. We denote the core radius as *R*_c_^sw^ and the outer radius of the microgel as *R* ^sw^, where the superscript “sw” indicates that the particle is in its swollen state. Various models have been proposed to describe the internal polymer density profile of core–shell microgels. A widely used approach represents the profile as the convolution of a homogeneous core with a Gaussian function, yielding a smooth radial decay of polymer density in the outer shell.^67,68^ In this work, we adopt an alternative representation based on a modified parabolic profile,^69,70^ in which the radial polymer number density ρ­(*r*) inside the microgel particle is given by

$$\rho \left(r\right)=\rho_{c}^{\mathrm{sw}}\{\begin{array}{ll} 1 & r\leq R_{c}^{\mathrm{sw}}\\ 1-\frac{{\left(r-R_{c}^{\mathrm{sw}}\right)}^{2}}{(R_{m}-R_{c}^{\mathrm{sw}})\left(R^{\mathrm{sw}}-R_{c}^{\mathrm{sw}}\right)} & R_{c}^{\mathrm{sw}}<r\leq R_{m}\\ \frac{{\left(R^{\mathrm{sw}}-r\right)}^{2}}{(R^{\mathrm{sw}}-R_{m})\left(R^{\mathrm{sw}}-R_{c}^{\mathrm{sw}}\right)} & R_{m}<r\leq R^{\mathrm{sw}}\\ 0 & r>R \end{array}$$ 2

here ρ_c_^sw^ represents the polymer density in the core (in the swollen state), and *R*_m_ ∈ [*R*_c_^sw^, *R* ^sw^] denotes an intermediate distance that controls the rate at which the density decays to zero in the shell. Figure 1 illustrates this density profile for different values of *R*_m_, assuming in all cases that the core radius is given by *R*_c_^sw^ = 0.6*R* ^sw^. This particular choice fairly well represents the density profiles of swollen cross-linked microgels obtained using SAXS.^63^ As shown, smaller values of *R*_m_ result in a faster decay of the density in the shell. When *R*_m_ = (*R* ^sw^ + *R*_c_^sw^)/2 = 0.8*R* ^sw^, the density profile in the shell becomes symmetrical.

**Figure 1:**
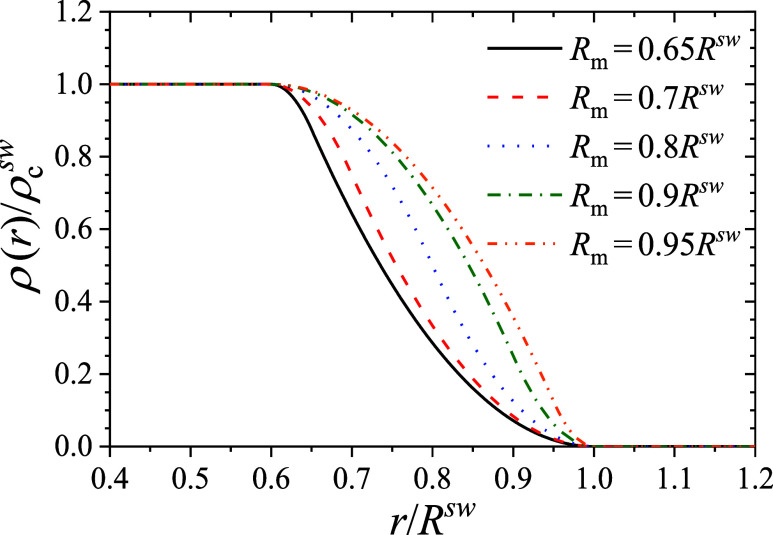
Polymer density inside a swollen microgel as a function of the distance to the center of the microgel, for different values of *R*_m_. In all cases, *R*_c_^sw^ = 0.6*R* ^sw^.

To facilitate analytical treatment, we adopted a simplified representation of the core–shell microgel by assuming that both the core and the shell possess constant polymer densities, as schematically illustrated in Figure 2. Under this assumption, the radial dependence of the polymer volume fraction can be approximated by

3 $$\phi \left(r\right)\approx \{\begin{array}{ll} \phi_{c}^{\mathrm{sw}} & r\leq R_{c}^{\mathrm{sw}}\\ \phi_{s}^{\mathrm{sw}} & R_{c}^{\mathrm{sw}}<r\leq R^{\mathrm{sw}},\\ 0 & r>R^{\mathrm{sw}} \end{array}$$

where ϕ_c_^sw^ and ϕ_s_^sw^ represent the average polymer volume fraction at core and shell in the swollen state, respectively. Both of them are obtained by integrating the density profile in different regions. Based on the parabolic density profile defined in eq 2 and denoting *v*_mon_ as the volume of a monomeric unit, the corresponding volume fractions can be derived analytically, leading to

$$\phi_{c}^{\mathrm{sw}}=\frac{3\upsilon_{mon}}{{\left(R_{c}^{\mathrm{sw}}\right)}^{3}}\int_{0}^{R_{c}^{\mathrm{sw}}}r^{2}\rho \left(r\right)\mathrm{dr}=\rho_{c}^{\mathrm{sw}}\upsilon_{mon}$$ 4

$$\phi_{s}^{\mathrm{sw}}=\frac{3\upsilon_{mon}}{{\left(R_{c}^{\mathrm{sw}}\right)}^{3}-{\left(R_{c}^{\mathrm{sw}}\right)}^{3}}\int_{R_{c}^{\mathrm{sw}}}^{R^{\mathrm{sw}}}r^{2}\rho \left(r\right)\mathrm{dr}=\frac{1}{10}\frac{\phi_{c}^{\mathrm{sw}}}{{\left(R^{\mathrm{sw}}\right)}^{3}-{\left(R_{c}^{\mathrm{sw}}\right)}^{3}}\left[{\left(R^{\mathrm{sw}}\right)}^{3}+{\left(R^{\mathrm{sw}}\right)}^{2}\left(R_{m}+R_{c}^{\mathrm{sw}}\right)+R^{\mathrm{sw}}\left(R_{m}^{2}+R_{m}R_{c}^{\mathrm{sw}}+{\left(R_{c}^{\mathrm{sw}}\right)}^{2}\right)+R_{m}^{3}+R_{m}^{2}R_{c}^{\mathrm{sw}}+R_{m}{\left(R_{c}^{\mathrm{sw}}\right)}^{2}-9{\left(R_{c}^{\mathrm{sw}}\right)}^{3}\right]$$ 5

**Figure 2:**
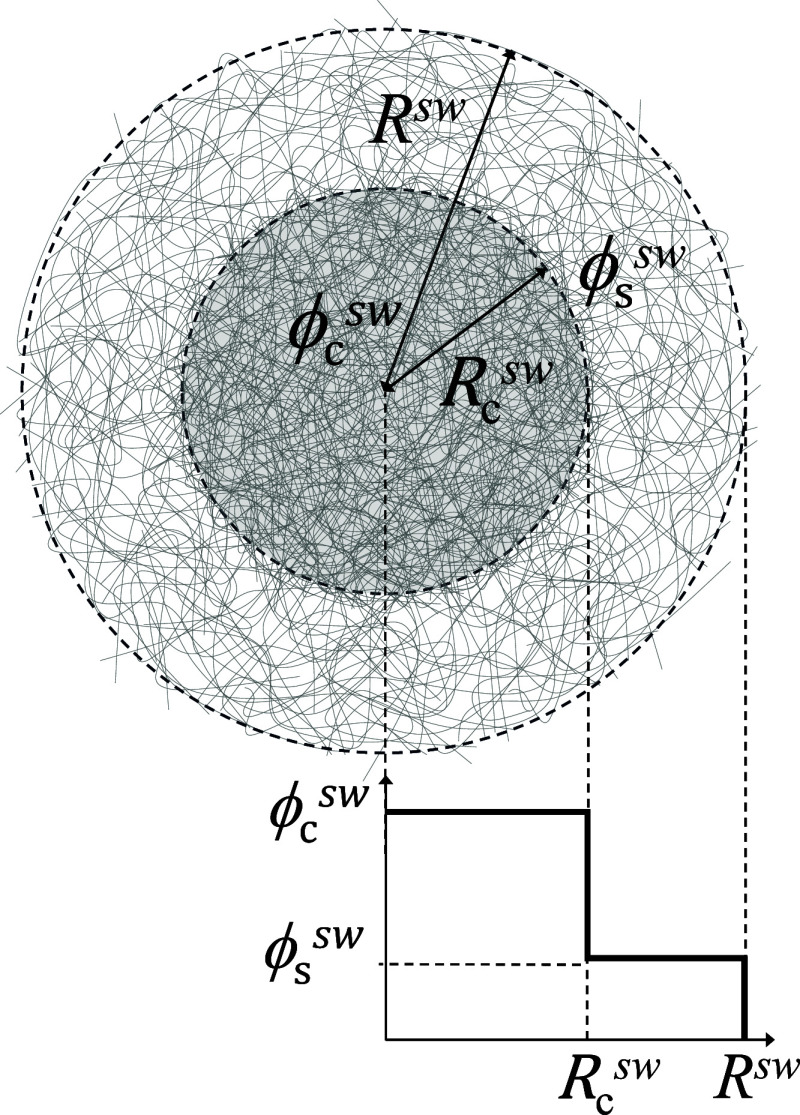
Schematic illustration of a core–shell microgel particle. In this representation, both the core and the shell are assumed to possess uniform polymer density distributions, characterized by volume fractions of *ϕ*_c_^sw^ and *ϕ*_s_^sw^, respectively.

In addition to the polymer volume fractions in the core and shell of a single swollen microgel, we can also estimate the average number of monomers per chain in both regions, denoted by *n*_c_ and *n*_s_, respectively. According to the c-theorem proposed by de Gennes,^71^ a swollen, cross-linked polymer network maintains a volume fraction ϕ that is proportional to the overlap polymer volume fraction ϕ* of an equivalent semidilute solution. This relationship is expressed as *ϕ*_*i*_^sw^ = *kϕ*_i_^*^ = *kn*_*i*_^–4/5^ (*i* = c, s) where *k* is a constant of order unity that depends on the cross-link functionality. This equation allows us to estimate *n*_*i*_ in both regions

6 $$n_{i}={\left(\frac{k}{\phi_{i}^{\mathrm{sw}}}\right)}^{5/4}\;\;\;i=c,s$$

### Flory–Rehner-Based Thermodynamics of a Heterogeneous Core–Shell Microgel

Having described the polymer distribution inside a microgel, the next step is to calculate the energy cost of compressing/expanding a single microgel, provided by the size-dependent intrinsic free energy of a single isolated microgel, *F*(*R*). Among all the possibilities, we chose the well-established Flory–Rehner theory, which has been shown to correctly model the thermoresponsive behavior of PNIPAM microgels.^62^

To this end, we first consider the particular case of a uniform microgel particle and then generalize the result to deduce the free energy for a microgel with a heterogeneous core–shell polymer distribution. Under this assumption, the microgel is composed of *N*_mon_ uniformly distributed interconnected monomeric units, which form polymer chains. These chains are connected by cross-linker molecules, with *N*_cross_ representing the total number of cross-linkers within the microgel, also uniformly distributed. For PNIPAM microgels, the total number of chains is given by *N*_ch_ = 2 *N*_cross_.^72^ Additionally, we define *n* as the average number of monomeric units per chain, given by *n* = *N*_mon_/*N*_ch_.

We consider that our thermoresponsive PNIPAM microgel is spherical with a radius *R* that depends on temperature. Assuming that the swelling of the particle is uniform, the polymer volume fraction inside the particle is given by ϕ = ϕ_0_(*R*_0_/*R*)^3^ where ϕ_0_ and *R*_0_ represent the polymer volume fraction and the radius in the reference (undeformed) state, corresponding to the conformation of the particle when it was synthesized. This synthesis is usually performed under bad solvent conditions, so the microgel is in the hydrophobic (collapsed) state.^72^ For PNIPAM microgels, the polymer network in the collapsed state is not a completely compact and dry structure, since some amount of water remains inside the pores between the cross-linked polymer chains. Consequently, the polymer volume fraction in this state is always lower than 1, i.e., ϕ_0_ < 1.

Within the Flory–Rehner theory, the intrinsic free energy of the microgel is split into three additive contributions: elastic, solvent-induced, and ionic free-energy terms

7 $$F=F_{\mathrm{elastic}}+F_{\mathrm{solvent}}+F_{\mathrm{ion}}$$

For the elastic free energy most authors make use of the popular rubber elasticity model^72^

8 $$F_{\mathrm{elastic}}\left(R\right)=\frac{3}{2}N_{\mathrm{ch}}k_{B}T\left[{\left(\frac{R}{R_{0}}\right)}^{2}-\ln\left(\frac{R}{R_{0}}\right)-1\right]$$

In eq 8, the elastic free energy includes a logarithm term introduced by Flory that accounts for the change in entropy when arranging the strands in the network.^73^ This contribution prevents the nonphysical collapse of the particle toward *R* → 0.

The solvent contribution to the free energy has an entropic part and a solvent–polymer interaction term^72,74–76^

9 $$F_{\mathrm{solv}}=k_{B}T\left(N_{\mathrm{ch}}\ln\phi +N_{\mathrm{solv}}\ln\phi_{\mathrm{solv}}+\chi N_{\mathrm{solv}}\phi \right)$$

where *N*_solv_ is the number of water molecules inside the microgel, whereas ϕ and ϕ_solv_ = 1 – ϕ are the corresponding volume fraction of polymer and water. Finally, χ is the Flory–Huggins parameter that controls the degree of solvent quality of the polymer chains.

The number of solvent molecules inside the microgel is given by *N*_solv_ = (1 – ϕ) *V*/*v*_solv_, where *V* is the volume of the microgel particle and *v*_solv_ is the volume occupied by a single water molecule. Similarly, the total number of monomers is *N*_mon_ = *nN*_ch_ = ϕ*V*/*v*_mon_. Unlike many previous studies in the literature, which approximate the volume of a monomeric unit as equal to that of a water molecule, we instead consider the more general situation, *v*_mon_ = *Bv*_solv_. Applying all these formulas and performing algebraic manipulations, the solvent contribution to the intrinsic free energy becomes

10 $$F_{\mathrm{solv}}=N_{\mathrm{ch}}k_{B}T\left[\ln\phi +\mathrm{nB}\left(\frac{1}{\phi }-1\right)\ln\left(1-\phi \right)+\mathrm{nB}\chi \left(1-\phi \right)\right]$$

The ionic contribution to the free energy arises only when the microgel contains a certain number of charged monomers, given by *fN*_ch_, where *f* represents the average number of charged monomers per polymer chain. In the absence of added salt, the number of counterions within the particle is also *fN*_ch_ in order to satisfy electroneutrality. By approximating the free energy of these ions as an ideal gas, the ionic free energy is

11 $$F_{\mathrm{ion}}=-k_{B}TN_{\mathrm{ch}}f\;\ln\left(V/V_{0}\right)$$

where *V*_0_ is the volume of the microgel in the reference (collapsed) state.

The final expression of the intrinsic free energy of the microgel, expressed in terms of ϕ, is

12 $$F\left(\phi \right)=N_{\mathrm{ch}}k_{B}T\left[\frac{3}{2}\left({\left(\frac{\phi_{0}}{\phi }\right)}^{2/3}-\ln{\left(\frac{\phi_{0}}{\phi }\right)}^{1/3}-1\right)+\ln\phi +\mathrm{nB}\left(\frac{1}{\phi }-1\right)\ln\left(1-\phi \right)+\mathrm{nB}\chi \left(T\right)\left(1-\phi \right)+f\;\ln\left(\frac{\phi }{\phi_{0}}\right)\right]$$

It should be noticed that due to the presence of charged monomers, an additional electrostatic free energy contribution depending on the effective net charge of the microgel (*Z*_net_) should be incorporated. However, in most situations of practical interest, the strong electrostatic attraction between the charged groups within the polymer network and their counterions greatly suppresses the number of counterions that actually leave the microgel.^77^ As a consequence, *Z*_net_ is typically several orders of magnitude smaller than the total nominal charge of the polymer network, making this electrostatic contribution to the free energy negligible in most cases.^72,78^

As shown, the free energy increases linearly with the total number of polymer chains in the microgel, *N*_ch_. Consequently, the microgel becomes stiffer as *N*_ch_ increases.

Despite the relatively large number of fitting parameters in eq 12, derived for a uniform microgel, the Flory–Rehner model has been shown to reproduce experimental data only with limited accuracy, mainly due to the heterogeneous internal distribution of polymer and cross-linkers within the particle.^79^ Therefore, the previous expression for the free energy must be generalized to account for the actual core–shell morphology, characterized by a dense core with a higher cross-linker concentration, surrounded by a more dilute shell. To properly describe the distinct properties of the core and shell, we express the total free energy as a sum of two contributions, corresponding to both regions, *F* = *F*_c_(ϕ_c_) + *F*_s_(ϕ_s_). Applying eq 12 independently to each region, we obtain

13 $$F_{i}\left(\phi_{i}\right)=N_{\mathrm{ch},i}k_{B}T\left[\frac{3}{2}\left({\left(\frac{\phi_{0i}}{\phi_{i}}\right)}^{2/3}-\ln{\left(\frac{\phi_{0i}}{\phi_{i}}\right)}^{1/3}-1\right)+\ln\phi_{i}+n_{i}B\left(\frac{1}{\phi_{i}}-1\right)\ln\left(1-\phi_{i}\right)+n_{i}B\chi \left(1-\phi_{i}\right)+f_{i}\ln\left(\frac{\phi_{i}}{\phi_{0i}}\right)\right],i=c,s$$

where *ϕ*_*i*_, *N*_ch,*i*_, *n*_*i*_, and *f*_*i*_ (with *i* = c, s) denote, respectively, the polymer volume fraction, the number of polymer chains, the average number of monomers per chain, and the average number of charged monomers per chain for each region. We also distinguish the polymer volume fraction in the collapsed state for the core and the shell, denoted as *ϕ*_0i_ (*i* = c, s), as it is expected to be lower for the shell. The corresponding sizes of the core and the full microgel in the collapsed state are denoted by *R*_0c_ and *R*_0_, respectively.

The osmotic pressure in each region is obtained as Π_*i*_ = −(1/*v*_solv_)­(∂*F*_*i*_/∂*N*_solv_)_*T*_ (*i* = c, s). Using that *ϕ*_*i*_ = *ϕ*_0*i*_ *V*_0*i*_/*V*_*i*_ = *ϕ*_0*i*_ *V*_0*i*_/(*V*_0*i*_ + *N*_solv_ *v*_solv_) and performing the partial derivative leads to

14 $$\Pi_{i}\left(\phi_{i}\right)=\frac{N_{\mathrm{ch},i}k_{B}T}{V_{0i}}\left[\left(\frac{3}{2}+f_{i}-n_{i}B\right)\frac{\phi }{\phi_{0i}}-{\left(\frac{\phi_{i}}{\phi_{0i}}\right)}^{1/3}-\frac{n_{i}B}{\phi_{0i}}\left(\ln\left(1-\phi_{i}\right)+\chi \left(T\right)\phi_{i}^{2}\right)\right],i=c,s$$

where *V*_0*i*_ (*i* = c, s) is the volume of core and shell regions in the reference collapsed state: *V*_0c_ = (4π/3)­(*R*_0c_^3^) and *V*_0s_ = (4π/3))­(*R*_0_^3^ – *R*_0c_^3^).

From the knowledge of χ­(*T*), the swelling state of the microgel as a function of temperature can be obtained by requiring that the osmotic pressure cancels in both regions, namely Π_c_(ϕ_c_) = 0 and Π_s_(ϕ_s_) = 0, which allows to calculate the equilibrium polymer volume fractions at this temperature, ϕ_c_^eq^(*T*) and ϕ_s_^eq^(*T*) respectively. The corresponding values of the core and outer microgel radii (*R*_c_(*T*) and *R*(*T*)) are then obtained from

15 $$R_{c}\left(T\right)=R_{0c}{\left(\frac{\phi_{0c}}{\phi_{c}^{\mathrm{eq}}\left(T\right)}\right)}^{1/3}$$

16 $$R\left(T\right)={\left[R_{c}^{3}+\frac{\phi_{0s}}{\phi_{s}^{\mathrm{eq}}\left(T\right)}\left({\left(R_{0}\right)}^{3}-{\left(R_{0c}\right)}^{3}\right)\right]}^{1/3}$$

### Mechanical Properties of Compressed Microgels

Before addressing the calculation of the interparticle interaction potential, we need to characterize the mechanical properties of the microgel. Responsive microgels have the ability to isotropically reduce their internal volume in response to mutual interactions. When this occurs, both the core and shell change their sizes. We remind that *R*_c_^sw^ and *R* ^sw^ are the radius of the core and the external shell in the swollen state for infinite dilute conditions, and ϕ_c_^sw^ and ϕ_s_^sw^ their corresponding volume fractions, respectively. For finite microgel concentrations, particle interactions reduce both sizes, becoming *R*_c_ and *R*. In this compressed state, the polymer volume fractions are denoted by ϕ_c_ and ϕ_s_. This compression does not occur symmetrically for the core and shell. Indeed, according to the experimental results, the loose shell is the region of the microgel becoming more compressed in response to small compressions, while the core size should be almost unaffected in this regime. The core size is expected to decrease only for sufficiently large compressions.

It is important to emphasize that in our model *R*_c_ and *R* are not independent variables, as they are connected by an additional constraint: the condition of mechanical equilibrium within the microgel. We consider that an external pressure *P* is applied uniformly distributed on its outer surface. This pressure originates from the repulsive interactions with other microgels in the suspension. By enforcing mechanical equilibrium, the osmotic pressures in both the core and shell must satisfy the following conditions

17 $$\Pi_{c}\left(\phi_{c}\right)=P,\Pi_{s}\left(\phi_{s}\right)=P$$

By numerically solving these coupled equations for increasing values of *P*, we obtained the corresponding polymer volume fractions in the core and shell of the compressed microgel (ϕ_c_ and ϕ_s_, respectively). Then, the values of *R*_c_ and *R* of the compressed microgel are given by

18 $$R_{c}=R_{c}^{\mathrm{sw}}{\left(\frac{\phi_{c}^{\mathrm{sw}}}{\phi_{c}}\right)}^{1/3}$$

19 $$R={\left[R_{c}^{3}+\frac{\phi_{s}^{\mathrm{sw}}}{\phi_{s}}\left({\left(R^{\mathrm{sw}}\right)}^{3}-{\left(R_{c}^{\mathrm{sw}}\right)}^{3}\right)\right]}^{1/3}$$

Please note that eqs 18 and 19 correlate both sizes and provide how *R*_c_ changes when *R* decreases. The reduction of *R* and *R*_c_ induced by microgel compression also entails a change of the intrinsic free energy of the particle, *F* = *F*_c_(ϕ_c_) + *F*_s_(ϕ_s_), which represents the energy cost associated with particle compression.

The next step is to characterize the mechanical properties of the core and shell for any compression state. We start with the bulk modulus, defined as^71^

20 $$K_{i}\left(\phi_{i}\right)=\phi_{i}{\left(\frac{\partial \Pi_{i}}{\partial \phi_{i}}\right)}_{T}$$

Performing the derivation, it leads to

21 $$K_{i}\left(\phi_{i}\right)=\frac{N_{\mathrm{ch},i}k_{B}T}{V_{0i}}\left[\left(\frac{3}{2}+f_{i}-n_{i}B\right)\frac{\phi }{\phi_{0i}}-\frac{1}{3}{\left(\frac{\phi_{i}}{\phi_{0i}}\right)}^{1/3}+\frac{n_{i}B}{\phi_{0i}}\left(\frac{\phi_{i}}{1-\phi_{i}}-2\chi \left(T\right)\phi_{i}^{2}\right)\right],i=c,s$$

The Young’s modulus of a cross-linked polymer network scales linearly with the density of chains as^80^

22 $$Y_{i}\left(R\right)\approx \frac{3}{2}k_{B}T\frac{N_{\mathrm{ch},i}}{V_{i}}\;\;\;i=c,s$$

where *V*_*i*_ (*i* = c, s) is the volume of the core and shell, respectively. This equation may be rewritten in terms of the polymer volume fraction, ϕ_*i*_ = *v*_mon_ *N*_ch,*i*_ *n*_*i*_/*V*_*i*_, as

23 $$Y_{i}=\frac{3}{2}k_{B}T\frac{\phi_{i}}{v_{\mathrm{mon}}n_{i}}\;\;\;i=c,s$$

Therefore, for a fixed *n*_*i*_ (i.e., at a constant cross-linker concentration), compressing the microgel leads to a linear growth of *Y*_*i*_ with ϕ_*i*_. Notably, the core constitutes a denser region with a higher concentration of cross-linkers, which leads to shorter average polymer chain lengths in comparison to those in the shell. As a consequence, the core exhibits a higher Young’s modulus, reflecting a stiffer elastic response relative to the softer, more loosely cross-linked shell.

Defining *Y*_c_^sw^ and *Y*_s_^sw^ as the Young moduli of the core and shell of an uncompressed swollen microgel, the corresponding moduli for any arbitrary compressed state can be obtained as

24 $$Y_{i}=\frac{\phi_{i}}{\phi_{i}^{\mathrm{sw}}}Y_{i}^{\mathrm{sw}}\;\;\;i=c,s$$

Finally, other relevant mechanical properties are Poisson’s ratios of the core and shell, denoted, respectively, as σ_c_ and σ_s_. For any compressed state, they are given by^65^

25 $$\sigma_{i}\left(\phi_{i}\right)=\frac{3K_{i}-Y_{i}}{6K_{i}}\;\;\;i=c,s$$

### Pair Interaction between Microgels

Having analyzed the scaling of the Young modulus in microgels with core–shell morphology, we are now ready to develop a model for their mutual interactions. To this end, we consider a system composed of *N* spherical microgels confined within a total volume *V*_T_. Throughout this analysis, we assume that the microgels are dispersed in a good solvent such that they remain in their fully swollen state.

In order to develop an interaction model, it is instructive to first consider a simpler scenario and assume that the full polymer network of the microgel is uniform. Under this assumption, the polymer density within a microgel with radius *R* is given by ρ = *N*_mon_/*V* = 3*N*_ch_ *n*/(4*πR* ^3^). Due to their responsive nature, microgels undergo thermal fluctuations in size around their equilibrium radius. Consequently, particles in the system may exhibit a distribution of radii, rendering the system intrinsically polydisperse.

In a good solvent, microgels with a uniform polymer density can be modeled as soft elastic spheres. When two such spheres of radii *R* and *R*′ come into contact and overlap (i.e., for interparticle distances *r* < *R* + *R*′), the resulting interaction is purely repulsive and can be described by an effective coarse-grained Hertzian pair potential^32,39,41,65^

26 $$\beta u\left(r;R,R^{\prime }\right)=\epsilon \left(R,R^{\prime }\right){\left(1-\frac{r}{R+R^{\prime }}\right)}^{5/2}\theta \left(R+R^{\prime }-r\right)$$

where θ­(*x*) denotes the Heaviside step function. This interaction potential accounts for the energetic cost associated with the interpenetration and elastic deformation of the microgels upon mutual overlap. The prefactor ϵ­(*R*, *R*′), expressed in units of *k*_*B*_ *T*, quantifies the strength of the repulsive interaction and is directly linked to the elastic properties of the cross-linked polymer networks comprising each microgel. In physical terms, ϵ­(*R*, *R*′) determines the effective hardness of the interaction: large values correspond to nearly hard-sphere-like behavior, while smaller values reflect softer interactions that permit significant overlap and deformation.

From elasticity theory, the repulsion strength ϵ­(*R*, *R*′) depends on the mechanical response of the interacting spheres.^41,65^ For notational convenience, we define the auxiliary function *A*(*R*) ≡ *Y*(*R*)/(1 – σ­(*R*)^2^), where *Y*(*R*) is the Young’s modulus and σ­(*R*) is the Poisson ratio of the microgel, both evaluated at radius *R*. The analytical expression of ϵ­(*R*, *R*′) is

27 $$\epsilon \left(R,R^{\prime }\right)=\frac{8}{15k_{B}T}A_{\mathrm{eff}}\left(R,R^{\prime }\right){\left(R+R\right)}^{\prime 2}{\left(\mathrm{RR}\right)}^{\prime 1/2}$$

where

$$A_{\mathrm{eff}}\left(R,R^{\prime }\right)=\frac{A\left(R\right)A\left(R^{\prime }\right)}{A\left(R\right)+A(R^{\prime })}$$ 28

The hardness of the interparticle potential can be estimated by evaluating the repulsive strength for equal-sized microgels, i.e., ϵ­(*R*, *R*). Taking into account the scaling behavior of the Young’s modulus *Y*(*R*), the interaction strength is given by

29 $$\epsilon \left(R,R\right)=\left(\frac{6}{5\pi }\right)\frac{N_{\mathrm{ch}}}{1-\sigma {\left(R\right)}^{2}}$$

As observed, the hardness exhibits a very weak dependence on particle size, with the only size-dependent contribution arising from the Poisson’s ratio. However, this result does not account for the experimental and simulation findings, which consistently show that the interparticle repulsion between microgels increases significantly upon compression. Clearly, the interaction model introduced in eq 26, based on a simple Hertzian potential and the assumption of a uniform polymer density, is insufficient to describe this behavior. The underlying limitation lies in the oversimplified assumption that the microgel has a homogeneous mass distribution.

To generalize this expression for microgels with a heterogeneous core–shell polymer distribution, we need to account for the mechanical properties of the two interacting microgels. We denote *R*_c_ and *R*_s_(≡ *R*) as the core and outer radii of a microgel in a given compressed state and notice that *R* is an independent variable, while *R*_c_ is constrained by the condition of internal mechanical equilibrium (cf. eq 17). Since the core and shell differ in cross-linker concentration and polymer density, they are characterized by distinct mechanical properties, namely, the Young’s moduli and Poisson’s ratios (*Y*_c_, σ_c_) for the core and (*Y*_s_, σ_s_) for the shell. To simplify the notation, we define *A*_c_ = *Y*_c_/(1 – σ_c_^2^) and *A*_s_ = *Y*_s_/(1 – σ_s_^2^).

The total interaction between two microgels retains the same Hertzian form but now includes four additive contributions corresponding to the core–core, core–shell, shell–core, and shell–shell Hertzian interactions. These contributions depend on the swelling state of both microgels and are incorporated in the following equation, where the superscript prime (′) denotes the second microgel

30 $$u\left(r\right)=u_{\mathrm{cc}}\left(r;R_{c},R_{c}^{\prime }\right)+u_{\mathrm{cs}}\left(r;R_{c},R_{s}^{\prime }\right)+u_{\mathrm{sc}}\left(r;R_{s},R_{c}^{\prime }\right)+u_{\mathrm{ss}}\left(r;R_{s},R_{s}^{\prime }\right)$$

where

31 $$\beta u_{\mathrm{ij}}\left(r;R_{i},R_{j}^{\prime }\right)=\epsilon_{\mathrm{ij}}\left(R_{i},R_{j}^{\prime }\right){\left(1-\frac{r}{R_{i}+R_{j}^{\prime }}\right)}^{5/2}\theta \left(R_{i}+R_{j}^{\prime }-r\right)$$

ϵ_*ij*_(*R*_*i*_, *R*_*j*_^′^) is given by

32 $$\epsilon_{\mathrm{ij}}\left(R_{i},R_{j}^{\prime }\right)=\frac{8C}{15k_{B}T}A_{\mathrm{eff},\mathrm{ij}}{\left(R_{i}+R_{j}^{\prime }\right)}^{2}{\left(R_{j}R_{j}^{\prime }\right)}^{1/2}\;\;\;i,j=c,s$$

where we introduced a proportionality constant *C* used to adjust the theoretical model for comparison with experimental or simulation data (as employed by Scotti et al.^41^). *A*_eff,*ij*_ values are given by

33 $$A_{\mathrm{eff},\mathrm{cc}}=\frac{A_{c}A_{c}^{\prime }}{A_{c}+A_{c}^{\prime }}-\frac{A_{c}A_{s}^{\prime }}{A_{c}+A_{s}^{\prime }}-\frac{A_{s}A_{c}^{\prime }}{A_{s}+A_{c}^{\prime }}+\frac{A_{s}A_{s}^{\prime }}{A_{s}+A_{s}^{\prime }}$$

34 $$A_{\mathrm{eff},\mathrm{cs}}=\frac{A_{c}A_{s}^{\prime }}{A_{c}+A_{s}^{\prime }}-\frac{A_{s}A_{s}^{\prime }}{A_{s}+A_{s}^{\prime }}$$

35 $$A_{\mathrm{eff},\mathrm{sc}}=\frac{A_{s}A_{c}^{\prime }}{A_{s}+A_{c}^{\prime }}-\frac{A_{s}A_{s}^{\prime }}{A_{s}+A_{s}^{\prime }}$$

36 $$A_{\mathrm{eff},\mathrm{ss}}=\frac{A_{s}A_{s}^{\prime }}{A_{s}+A_{s}^{\prime }}$$

It is important to emphasize that the different contributions appearing in eqs 33–36 account for the need to subtract the contribution of the internal hole to avoid overcounting the pair interactions (see the illustration in Figure 3, where this decomposition into nine positive and negative contributions is depicted schematically). Interestingly, these expressions satisfy the consistency condition, ensuring that the multi-Hertzian potential reduces to a single Hertzian term when both interacting microgels share identical mechanical properties, i.e., when *A*_c_ = *A*_s_ and *A*_c_^′^ = *A*_s_^′^.

**Figure 3:**
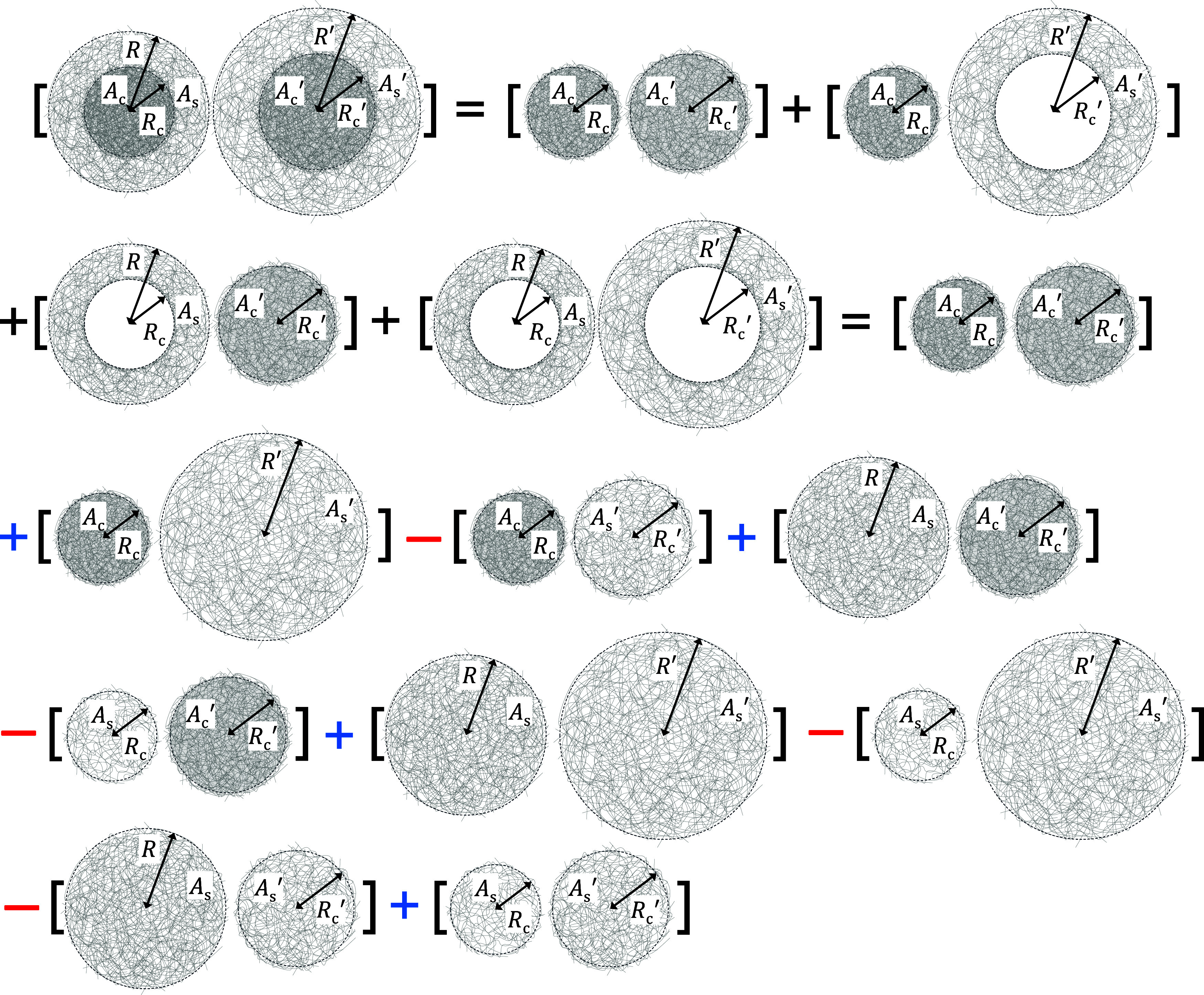
Scheme of the decomposition of the pair interaction between two microgels into Hertzian pair potentials. Negative terms are corrections needed to avoid overcounting pair interactions.

As observed, the final resulting pair interaction between dissimilar microgels necessarily requires four Hertzian contributions. This outcome agrees with previous studies that use coarse-grained simulations to explain the structural and dynamical behavior of binary mixtures of microgels in the swollen state, using a nonresponsive phenomenological multi-Hertzian pair potential with precisely four additive terms.^66^ Thus, we demonstrate that the use of four Hertzian contributions arises from the core–shell internal morphology of the microgels, which deform differently upon contact due to the disparity in Young’s moduli between the core and shell regions. Additionally, responsiveness allows the particles to undergo isotropic compression due to interactions modeled by the previously described Flory–Rehner internal free energy, enabling particle size fluctuations around the mean.

## Experimental: Determination of Model Parameters Based on Synthesized PNIPAM Microgel Properties

After establishing the theoretical model, we proceed to extract the relevant parameters by applying it to a real experimental system using PNIPAM microgels as a reference. These microgels were synthesized in Milli-Q water via precipitation polymerization, following the protocol detailed in a previous study.^81^ *N*-isopropylacrylamide (NIPAM) was used as the monomer, *N*,*N*’-methylene-bis­(acrylamide) (BIS) as cross-linker, and potassium persulfate (KPS) as initiator, with a 4.8% mol cross-linking density. All reagents were purchased from Sigma-Aldrich and used as received. As a result of this synthesis process, the cross-linking density follows a Gaussian profile, featuring a more densely cross-linked core and a less cross-linked shell. Based on the synthesis data and the calculation of the number of particles in the suspension obtained using nanoparticle tracking analysis (NTA), we estimate the average number of monomers per chain as *n* = *N*_mon_/*N*_ch_ ≈ 8.95. The PNIPAM microgels carry a slight charge due to the initiator used during particle synthesis, resulting in an average number of charged monomers per chain of *f* = 0.107. This low degree of charging allows the microgel to be treated effectively as a weakly charged particle, thereby justifying the omission of the electrostatic contribution to the free energy.

The hydrodynamic radii of the microgel in its swollen and collapsed states were experimentally determined via dynamic light scattering (Malvern Zetasizer NanoZ), yielding *R* ^sw^ = (345 ± 25) nm at *T* = 15 °C and *R*_0_ = (200 ± 10) nm at *T* = 48 °C. The corresponding core radius in the swollen state is estimated as *R*_c_^sw^ = 0.6*R* ^sw^ = (207 ± 15) nm. It is worth noting that microgel sizes determined by DLS are typically slightly larger than those obtained by small-angle neutron scattering (SANS). This discrepancy is attributed to a small number of dangling polymer chains at the particle surface, which affect the hydrodynamic radius measured by DLS but are present in concentrations too low to be detected by SANS.^67^ Given the relatively minor difference between both techniques, we will consider the DLS measurements as representative of the particle size throughout this work.

The full swelling behavior of the thermoresponsive PNIPAM microgels obtained through DLS is shown as square symbols in Figure 4(a), which plots the mean hydrodynamic radius as a function of the temperature. We use as a reference the microgel radius in the swollen state, namely, *R* ^sw^ = 345 nm (*T* = 15 °C), to normalize the experimental data. As observed, the particle size exhibits the expected behavior: at low temperatures, the microgels are in a swollen state, with the size reaching a plateau well below the transition temperature. As the temperature increases, the size decreases, eventually stabilizing at a new plateau corresponding to the collapsed state. The experimental data are well described by a sigmoid function, shown as a solid line in Figure 4(a)

37 $$\frac{R\left(T\right)}{R^{\mathrm{sw}}}=\delta_{0}+\frac{\delta_{1}-\delta_{0}}{1+\exp\left(\left(T-T_{\mathrm{LCST}}\right)/\Delta \right)}$$

where δ_1_ = (1.0073 ± 0.0043), δ_0_ = *R*_0_/*R* ^sw^ = (0.580 ± 0.003), *T*_LCST_ = (30.57 ± 0.12) °C and Δ = (3.43 ± 0.09) °C. The resulting LCST transition temperature is close to the reported value for PNIPAM microgels.^41,79,82^

**Figure 4:**
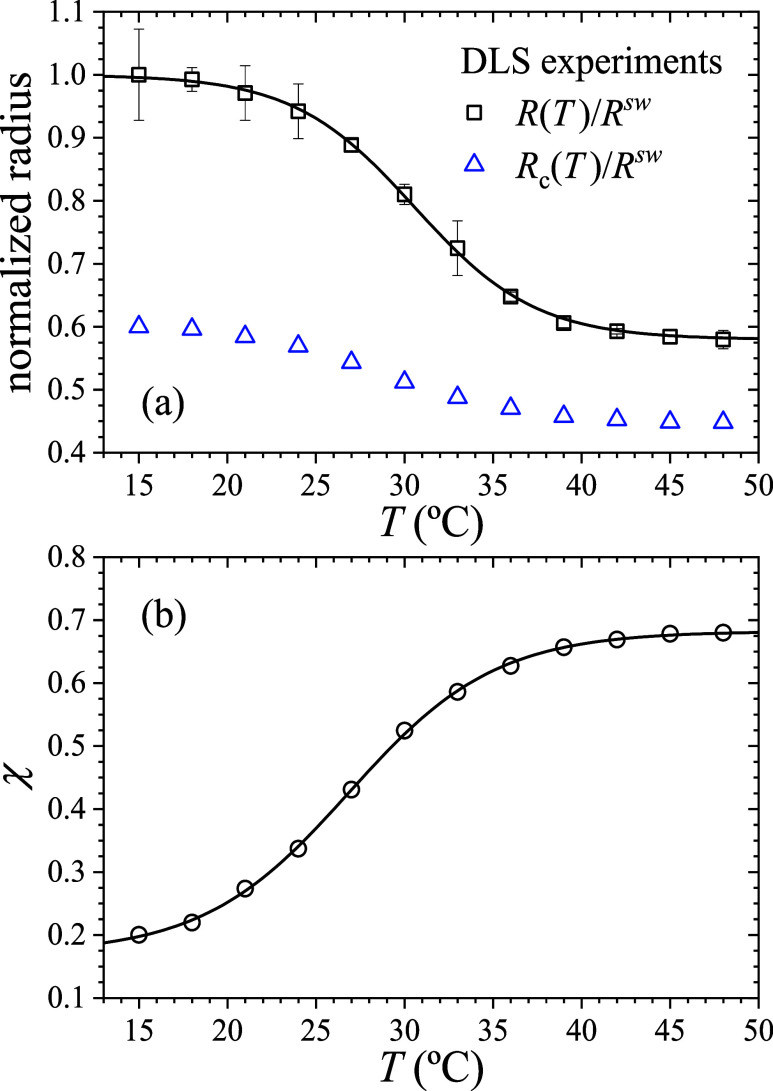
(a) Squares: normalized microgel radius (*R*/*R* ^sw^) as a function of temperature obtained experimentally from DLS. Solid line: fitting of the experimental data using the sigmoidal function given by eq 37. Blue triangles: normalized radius of the internal core (*R*_c_/*R* ^sw^) as a function of *T*. (b) Circles: Temperature dependence of the Flory solvency parameter was obtained by solving Π_c_ = Π_s_ = 0 (eq 21). Solid line: fitting of χ­(*T*) using eq 41.

It is interesting to measure the radius in the fully dry state, so we first functionalized a silicon substrate (⟨100⟩ orientation, p-type, Boron-doped, 1–10 Ω cm, University Wafer Inc., United States) with (3-Aminopropyl)­triethoxysilane (APTES ≥ 98.0%, Sigma-Aldrich) by immersion in a 1:1 solution of APTES and isopropanol for 30 min under gentle shaking. Next, we ensured the bonding by placing the substrate on a hot plate at 100 °C for 1 min, rinsing with isopropanol afterward. This makes the substrate positively charged, and the microgels adhere via electrostatic interactions from dispersion. After this, we kept the microgel-laden substrate wet and exchanged the solvent by Milli-Q water, ethanol, and acetone. Then the substrate is subjected to CO_2_ supercritical drying (Polaron CPD 7501) to avoid the capillary forces that deform the microgels upon drying from any liquid. In this way, we avoid the deformation of the microgels due to drying effects, instead showing a half sphere morphology due to the sticking of the microgel to the substrate and the conformation of the dry polymeric network. Finally, we characterized the fully dry minimum radius by means of Atomic Force Microscopy (Dimension 3000), yielding *R*_min_ = (171 ± 13) nm, corresponding to the average half-maximum height of 68 microgels. This is in good agreement with the estimation of the radius of equivalent spheres corresponding to the volume of each microgel, (174 ± 10) nm in our case. From these values, the polymer volume fraction in the collapsed state is ϕ_0_ = (*R*_min_/*R*_0_)^3^ = 0.625, which falls within the range of values typically reported in the literature for PNIPAM microgels.^82–84^ The parameter *B* = *v*_mon_/*v*_s_ is also required as it explicitly enters the expressions for both the free energy and the osmotic pressure. The volume of a water molecule under normal conditions is *v*_s_ = 0. 03 nm^3^. The volume of a NIPAM monomer can be estimated from its molecular weight (*M*_W_ = 113.16 g/mol), density (ρ_m_ ≈ 1.1 g/cm^3^), and assuming that the polymer forms an amorphous solid phase with a polymer volume fraction given by the random close packing as *v*_mon_ = 0.64 M_W_/(ρ_m_ *N*_A_) = 0.1093 nm^3^. Hence, *B* = 3.644.

Experimental studies on the polymer distribution in swollen microgels suggest that the decay in polymer density is more pronounced in the inner region of the shell (i.e., for distances *r* close to *R*_c_), while the low-density tail smoothly extends to zero in the outer region of the shell.^63^ To capture this behavior, we propose that *R*_m_= 0.65*R* ^sw^. With this particular choice, the average polymer volume fraction in the shell (calculated through eq 5) is ϕ_s_^sw^ = αϕ_c_^sw^, with α = 0.286. Using eq 6 to eliminate *n*_c_ and *n*_s_ in eq 23 provides the relation between the core and shell Young moduli of the microgel in the swollen state, as *Y*_c_^sw^ = α^–9/4^ *Y*_s_^sw^ = 16.75*Y*_s_^sw^.

Then, the number of chains in the core and shell domains is obtained as

38 $$N_{\mathrm{ch},s}=\frac{8\pi Y_{s}^{\mathrm{sw}}}{9k_{B}T}\left({\left(R^{\mathrm{sw}}\right)}^{3}-{\left(R_{c}^{\mathrm{sw}}\right)}^{3}\right),N_{\mathrm{ch},c}=\frac{8\pi Y_{c}^{\mathrm{sw}}}{9k_{B}T}{\left(R_{c}^{\mathrm{sw}}\right)}^{3}$$

which allows the calculation of the fraction of chains in the core

39 $$x=\frac{N_{\mathrm{ch},c}}{N_{\mathrm{ch}}}=\frac{{\left(R_{c}^{\mathrm{sw}}\right)}^{3}}{{\left(R_{c}^{\mathrm{sw}}\right)}^{3}+\alpha^{9/4}\left({\left(R^{\mathrm{sw}}\right)}^{3}-{\left(R_{c}^{\mathrm{sw}}\right)}^{3}\right)}=0.822$$

The average number of monomers per chain in the core and shell can also be calculated. On the one hand, using eq 6 we find that *n*_c_/*n*_s_ = (ϕ_s_/ϕ_c_)^5/4^ = α^5/4^ = 0.209. On the other hand, *n*_c_ *x* + *n*_s_(1 – *x*) = *n* = 8.95. Solving this system of equations leads to *n*_c_ = 5.35 and *n*_s_ = 25.59.

Regarding the distribution of the charged group monomers within the microgel originated from the initiator used during synthesis, we assume that are entirely localized in the outer shell of the microgel. Accordingly, we set *f*_c_ = 0 and *f*_s_ = *f*/(1 – *x*) = 0.601. For the polymer packing fractions in the reference collapsed state, we consider ϕ_0c_ = ϕ_0_ = 0.625 for the core and ϕ_0s_ = 0.433ϕ_0_ = 0.271 for the shell. With this choice, the resulting polymer volume fractions in both phases in the swollen state (obtained imposing the conditions Π_c_ = Π_s_ = 0 with χ = 0.2, as we l will see later) are ϕ_c_^sw^ = 0.153 and ϕ_s_^sw^ = 0.044. We note that these values satisfy the relation ϕ_s_^sw^ = 0.286ϕ_c_^sw^ imposed before. The radius of the inner core in the collapsed state is *R*_0c_ = *R*_c_^sw^(ϕ_c_^sw^/ϕ_0c_)^1/3^ = 129 nm, whereas the corresponding values of the volume of the core and shell in the collapsed state are *V*_0c_ = (4π/3) *R*_0c_^3^ = (4π/3)­(ϕ_c_^sw^/ϕ_0c_)­(*R*_c_^sw^)^3^ = 9.06 × 10^6^ nm^3^ and *V*_0s_ = (4π/3)­(*R*_0_^3^ – *R*_0c_^3^) = 2.17 × 10^7^ nm^3^.

To match the repulsion strengths reported by Del Monte and Zaccarelli for swollen microgels,^49^ we set ϵ_ss_ = 470 (in units of *k*_*B*_ *T*). Additionally, in order to reproduce the particle volume fractions observed in their simulations of compressed systems, we used a value of *C* = 30. This indicates that the microgel is more deformable than would be expected based solely on this interparticle repulsion. A possible explanation for this enhanced deformability is the large flexibility of the outer region of the microgel shell, formed by dangling chains that are sparsely cross-linked. The low cross-linker density in this region allows greater chain deformation, which is not captured in our theoretical model, where the average cross-linker concentration is assumed to be uniform throughout the shell. Within the framework of our approximate theory, the only way to account for this effect is by introducing this constant *C*, effectively increasing the particle compressibility by a factor of 30.

With these parameter choices, and using eqs 32 and 36, we estimate the Young’s modulus of the outer shell in the swollen state as $Y_{s}^{\mathrm{sw}}=\frac{15}{16C}\frac{\epsilon_{\mathrm{ss}}k_{B}T\left(1-\sigma_{s}^{2}\right)}{{\left(R^{\mathrm{sw}}\right)}^{3}}=1.15\mathrm{Pa}$ , and that of the inner core as *Y*_c_^sw^ = 19.3 Pa. For the rest of the interaction strengths between swollen microgels, we find that 2ϵ_cs_ = 414 and ϵ_cc_ = 1418. It should be noted that ϵ_cc_ ≈ 3ϵ_ss_, which agrees with the results reported in ref 49. This confirms that the enhanced repulsion between the cores is a direct consequence of the larger cross-linker concentration in that region. The estimation of the number of polymer chains in both regions, as provided by eq 38, yields *N*_ch,s_ = 26.2 and *N*_ch,c_ = 120.4, which will be used to calculate the intrinsic free energy of the core and shell, respectively.

The Young’s modulus values obtained in our theoretical approach are notably lower than typical experimental measurements. This discrepancy arises because the model parameters were adjusted to match the repulsion strengths used in in silico microgel suspensions reported in ref 49, of about 500 *k*_*B*_ *T*. These parameters were originally developed for smaller microgels, with radii around 50 nm and about 150–200 polymer chains, for which Young’s moduli typically range from 10^2^ to 10^4^ Pa, in good agreement with experimental observations.^85^ Applying these parameters to larger, experimentally relevant microgels results in a much lower density of network chains and, consequently, reduced mechanical stiffness. While this does not affect the predicted swelling behavior, which is determined by the osmotic pressure balance between the core and shell, it does influence the mechanical response. Specifically, increasing the number of chains enhances effective repulsions and resistance to deformation, leading to higher Young’s modulus values. In this context, the relatively low moduli obtained may reflect the application of a parameter regime appropriate for smaller microgels rather than a shortcoming of the theoretical framework itself. Adjusting the interaction strength to account for system size, or considering its scaling more explicitly, could help refine the model’s predictive power for mechanical properties without altering its thermodynamic consistency. Indeed, considering microgels with a swollen radius of approximately 50 nm, the shell Young’s modulus obtained assuming ϵ_ss_ = 470 is *Y*_s_^sw^ = 380 Pa, which is fully consistent with experimental measurements on PNIPAM water-swollen hydrogels obtained via scanning force microscopy.^85^ The core Young’s modulus in the swollen state is *Y*_c_^sw^ = 6352 Pa, reflecting the stiffer mechanical response of the core, while in the collapsed state it becomes *Y*_c0_ = *Y*_c_^sw^ (*R*_c_^sw^/*R*_c0_)^3^ = 26.2 kPa, also in agreement with experimental data. These results demonstrate that, when using particle sizes consistent with the experimental system, the predicted mechanical properties of both the shell and core are coherent with observations.

## Computer Simulations

Having established the theoretical framework for an isolated core–shell microgel particle and its pairwise interactions by means of a multi-Hertzian potential, we now turn to the study of fluids composed of these particles at varying packing densities. This analysis reveals emergent collective behaviors that cannot be inferred solely from the properties of individual particles or their binary interactions.

To this end, we have performed Monte Carlo (MC) simulations in the NPT ensemble using cubic simulation boxes with periodic boundary conditions containing *N* = 1000 spherical particles that interact via the multi-Hertzian potential given in eqs 30–33. The reduced temperature is fixed at T* ≡ *T*/*T*_LCST_ = 0.9487, whereas the reduced pressure, defined as P* ≡ 8­(*R* ^sw^)^3^ *P*/(*k*_*B*_ *T*_LCST_), is varied over a broad range from 0.05 to 13000. Each MC cycle consisted of *N* trial moves, in which a randomly selected microgel (particle μ) was either displaced (**r**_μ_ → **r**_μ_ + Δ**r**_μ_) or had its size changed (*R*_μ_ → *R*_μ_ + Δ*R*_μ_), along with an additional attempt to modify the box volume (*V*_T_ → *V*_T_ + Δ*V*). Particle moves were selected randomly, with 80% probability assigned to displacements and 20% to size changes. This combination was chosen after evaluating the efficiency of different moving sets in dilute and concentrated systems. All moves—including displacements, swelling/deswelling, and volume changes—were accepted or rejected according to the standard Metropolis algorithm, using the Hamiltonian

40 $$H=\sum_{\mu <\nu =1}^{N}u\left(\left|r_{\mu }-r_{\nu }\right|;R_{\mu },R_{\nu }\right)+\sum_{\mu =1}^{N}F\left(R_{\mu }\right)+PV_{T}$$

where *F*(*R*_μ_) is the intrinsic free energy of particle μ as provided by eq 13, and *u*(|**r**_μ_ – **r**_ν_|; *R*_μ_, *R*_ν_) is the interaction pair potential between particles μ and ν given in eqs 30–36. Note that for both particle size and volume changes, the variation in interaction energy must also be evaluated, in addition to the free energy associated with the size and the change in *PV*_T_, as appropriate.

Simulations typically required up to 2 × 10^6^ MC cycles to reach equilibrium, as determined by the stabilization of key observables such as the packing fraction and the energy of the system. Initial configurations were generated randomly at very low density, with spherical particles distributed isotropically throughout the simulation box. The particle sizes were drawn from parent distribution *p*(*R*). At such a low density, the particle radius naturally adopted a value close to the minimum of the free energy, approximately *R* ^sw^. Once equilibrium was achieved, a production run of 2 × 10^6^ additional MC cycles was carried out to compute the observables of interest, including particle size distributions and radial distribution functions.

## Results and Discussion

### Testing the Free Energy Model to Describe the Thermal Collapse of the Microgel

The theoretical model presented earlier decomposes the microgel’s intrinsic free energy into Flory–Rehner-based core and shell contributions (cf. eq 13) and yields expressions for the osmotic pressures in both regions (eq 21). We now turn to evaluating the model’s ability to reproduce experimental data on thermal collapse.

With this aim, we determine the values of the Flory solvency parameter, χ, that reproduce the experimentally observed swelling behavior. For this purpose, we apply internal mechanical equilibrium conditions in core and shell, i.e., Π_c_ = 0 and Π_s_ = 0 (see eq 21) for each radius *R* measured via DLS. The resulting values of χ are plotted as square symbols in Figure 4(b). As expected for PNIPAM microgels, χ increases with temperature, reflecting the increasing hydrophobicity of the polymer chains above the LCST, which drives the deswelling process. The obtained values of χ lie within the range of commonly accepted values, confirming that the theoretical model provides a reliable description of the system. To analytically reproduce the extracted values of χ, we fit the data using an empirical sigmoid function, given by

41 $$\chi \left(T\right)=\chi_{1}+\frac{\chi_{0}-\chi_{1}}{1+\exp\left(-\kappa \left(T-T_{\chi }\right)\right)}$$

The fit yields the following parameter values: χ_1_ = 0.170 ± 0.005, χ_0_ = 0.682 ± 0.003, κ = (0.242 ± 0.007) °C^–1^, and *T*_χ_ = (26.86 ± 0.13) °C. The extracted crossover temperature *T*_χ_ slightly underestimates the experimental lower critical solution temperature, *T*_LCST_. We attribute this deviation to the fact that χ was fitted solely as a function of temperature, in contrast to other models that also expand χ as a power series of the microgel volume fraction.^84^

Employing the core–shell intrinsic free energy model not only provides an accurate fit of the overall thermal collapse of the microgel but also offers a key advantage: it captures the temperature-dependent collapse of the microgel core, *R*_c_(*T*), as evidenced by the blue triangles in Figure 4(a). As observed, the core radius is *R*_c_ = 0.6*R* ^sw^ at low temperatures, and decreases to its collapsed value of *R*_c_ = 0.45*R* ^sw^ for temperatures above the LCST. It is worth noting that the core undergoes a less pronounced deswelling compared to the entire microgel, with *R*_c_(48 °C)/*R*_c_(15 °C) = 0.75 versus *R*(48 °C)/*R*(15 °C) = 0.58. This reduced shrinkage of the core is attributed to its higher cross-linker concentration, which limits its swelling capacity.

### Reduction of Core Size Induced by Mechanical Compression

Having analyzed the complete swelling response as a function of the temperature, we now focus on the specific case of swollen microgels. To this end, we fix the temperature at *T* = 15 °C, corresponding to χ = 0.2.

To fully characterize the compression of an initially swollen microgel induced by particle interactions, we need the variation of the radius of the internal core for different compression states, i.e., *R*_c_(*R*). This dependence can be obtained imposing internal mechanical equilibrium Π_c_ = Π_s_ = *P*, for different applied external pressures onto the microgel, *P* (see eqs 17–19). The results are shown in Figure 5, which depicts the dependence of *R*_c_/*R* ^sw^ as a function of *R*/*R* ^sw^. Please note that values of *R*_c_/*R* ^sw^ > 1 correspond to situations where the microgel is stretched outward by applying negative external pressures (*P* < 0). As shown, under small compressions, *R* decreases, while the core radius remains nearly unchanged, reflecting the greater stiffness of the core compared to the shell. However, as the applied pressure increases, the system enters a different mechanical regime in which *R*_c_ also undergoes a significant reduction. The full compression behavior can be accurately described by the following empirical expression

42 $$\frac{R_{c}}{R^{\mathrm{sw}}}=A_{1}{\left[1-A_{2}\exp\left(-{\left(\frac{R/R^{\mathrm{sw}}}{A_{3}}\right)}^{A_{4}}\right)\right]}^{1/A_{4}}$$

with *A*_1_ = 0.604, *A*_2_ = 1.05, *A*_3_ = 0.712, and *A*_4_ = 4.06.

**Figure 5:**
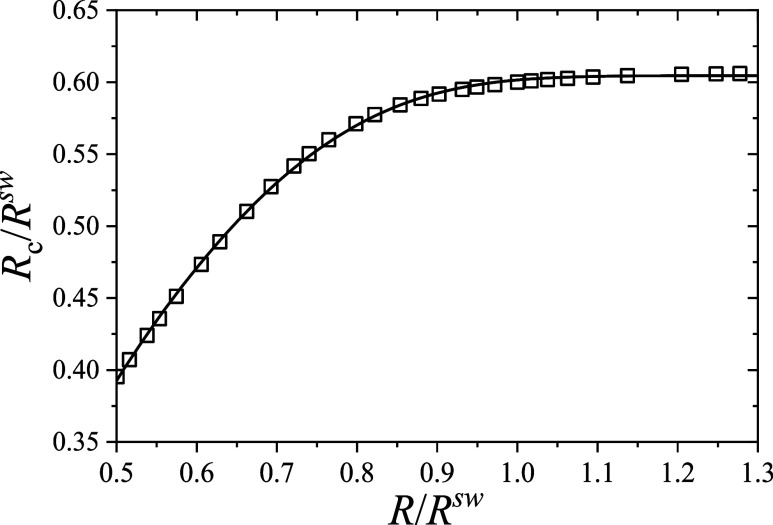
Symbols: Core radius of a compressed microgel as a function of its external radius *R*_c_ = *R*_c_(*R*) obtained by solving eqs 17–19. The results are normalized by the external radius of the microgel in the swollen state, *R* ^sw^. Solid line: fitting of the results using the empirical expression given in eq 42.

### Intrinsic Free Energy of a Swollen Microgel

Once the dependence *R*_c_(*R*) has been obtained, we are now able to compute the total intrinsic free energy of a single microgel as a function of the microgel radius, *F*(*R*) = *F*_c_(*R*) + *F*_s_(*R*). Figure 6(a) displays *F* as a function of the normalized radius *R*/*R* ^sw^ for χ = 0.2. For this value of the Flory parameter, the microgel is in its swollen state, and as expected, *F*(*R*) exhibits a minimum at *R*/*R* ^sw^ = 1. This corresponds to the equilibrium size in the absence of external forces. Departures from this minimum lead to the emergence of an intrinsic restoring force, given by −(∂*F*/∂*R*)_*T*_, which tends to return the particle to its equilibrium size.

**Figure 6:**
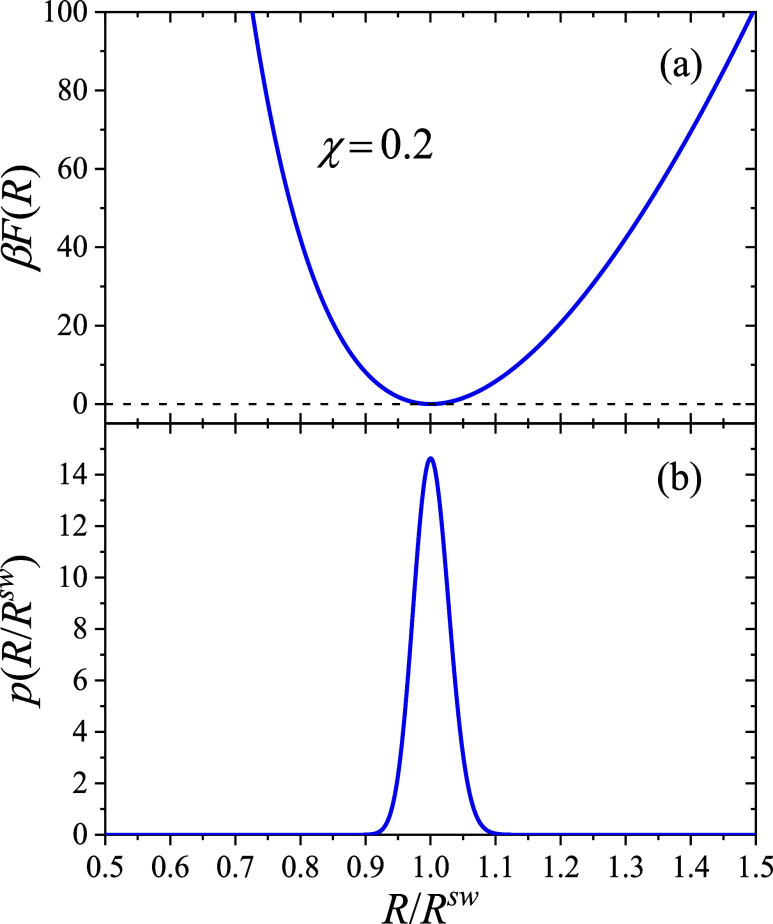
(a) Total intrinsic free energy of a single microgel as a function of the outer radius, *F*(*R*), calculated for χ = 0.2 (swollen state). (b) Parent probability size distribution, *p*(*R*), of the responsive microgels for infinitely diluted suspensions.

Figure 6(b) shows the resulting parent size distribution, defined in eq 1, representing the size probability distribution of a microgel in the infinite dilution limit, where interparticle interactions can be neglected. As expected, *p*(*R*) attains its maximum at *R* = *R* ^sw^. It is important to highlight that microgel responsiveness inherently gives rise to a polydisperse size distribution even in the absence of interactions. In more concentrated suspensions, repulsive interparticle interactions compress the microgels, thereby modifying the equilibrium size distribution to a new function, *f*(*R*), whose peak is expected to shift toward smaller sizes. This effect will be discussed in greater detail in the following section.

After fixing all the physical parameters involved in the theoretical model and characterizing *R*_c_(*R*), the values of the interaction strengths between any pair of microgels in different compression states (ϵ_*ij*_) are calculated using eq 32, the Young’s moduli computed by means of eq 24 and the Poisson’s ratio obtained from eq 25 (leading to values that range from σ = 0.43 in the swollen uncompressed state to σ = 0.5 in the more collapsed state). In the following section, the obtained ϵ_*ij*_ parameters are employed in Monte Carlo simulations to examine how the structural properties of the microgel suspension evolve with increasing particle density.

The data described in this section, obtained from fitting the theory to the experimental results, are summarized in part in Table 1, with special attention to those used in the computer simulations.

### Structural Properties of Simulated Suspensions of Swollen Microgels under Compression

In the following, we discuss the physical collective properties of a suspension of microgels in the swollen state (χ = 0.2) obtained from MC simulations for different compression states. For this purpose, we vary the reduced pressure of the system from P* = 0.05 to 13000. For each equilibrium configuration we calculate the corresponding nominal volume fraction, ζ, defined as the volume fraction the system would exhibit if all particles retained their fully swollen size at infinite dilution, i.e., with a probability size distribution given by the parent *p*(*R*). This quantity is given by

43 $$\zeta =\frac{4}{3}\pi \frac{N}{V_{T}}\int R^{3}p\left(R\right)dR$$

where *V*_T_ is the volume of the simulation box. Snapshots from MC simulations, illustrating the progressive packing and structural ordering of microgel particles as the nominal volume fraction increases from ζ = 0.49 to 9.52, are shown in Figure 7.

**Figure 7:**
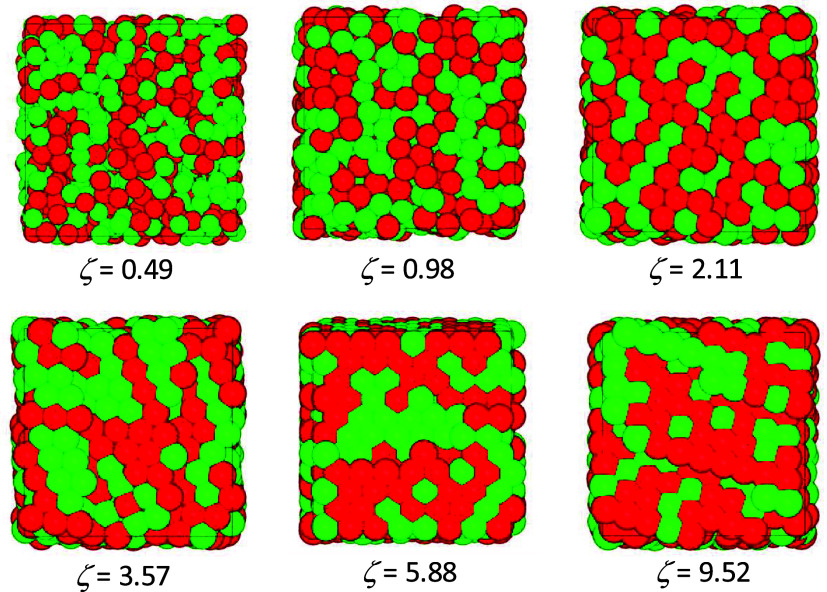
Snapshots of the microgel suspension obtained by MC simulations for increasing nominal packing fractions, from ζ = 0.49 (top left) to 9.52 (bottom right). Red and green colors indicate particle sizes larger and smaller, respectively, than the mean size.

Unlike the nominal volume fraction, the effective volume fraction η reflects the instantaneous particle sizes, thus capturing the system’s inherent size polydispersity. This quantity can be readily computed within our responsive model as follows

44 $$\eta =\frac{4}{3}\pi \frac{N}{V_{T}}\int R^{3}f\left(R\right)dR$$

where *f*(*R*) is the actual size distribution at this compression state. It is important to distinguish both ζ and η from polymer volume fraction ϕ, defined in eq 3. While ϕ characterizes the internal polymer density within individual microgel particles, ζ and η describe the packing behavior of the particle suspension as a whole.

As the system is compressed, the pressure-induced reduction in the particle size leads to progressively greater spatial overlap between neighboring particles. This behavior, already evident in the snapshots of Figure 7, is quantified in Figure 8, which shows the dependence of the effective volume fraction η on the nominal volume fraction ζ, in good agreement with previous reports.^22,46,49,86^ The condition η = ζ (depicted as a black dashed line in Figure 8) characterizes a nonresponsive system, in which particle compression is negligible. In the dilute regime, η and ζ coincide, as particles remain effectively isolated and adopt their equilibrium size by minimizing the intrinsic free energy described in eq 13. At higher concentrations, however, interparticle interactions become significant. To reduce mechanical contact and overlap, particles shrink, leading to systematic deviations from the nonresponsive behavior η = ζ.

**Figure 8:**
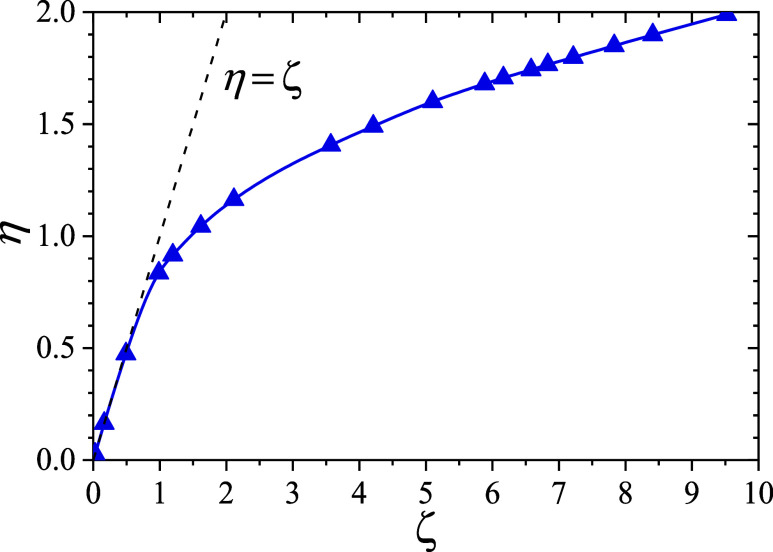
Dependence of the effective particle volume fraction η (calculated from eq 44 using the size distribution obtained from MC simulations for this particle concentration, *f*(*R*)) as a function of the nominal volume fraction ζ (calculated assuming the size distribution in diluted conditions, *p*(*R*)). The dashed line η = ζ shows the behavior for nonresponsive microgels, while the solid line serves as a guide to the eye. In all cases at T* = 0.9487 (i.e., *T* = 15 °C).

Our results indicate that particle interpenetration becomes relevant when the average center-to-center distance between neighboring particles, as obtained from the first peak of the radial distribution function, falls below the average particle diameter. This occurs at ζ ≈ 0.31, which we consider to be an approximate lower bound for the onset of overlap. Due to the system’s polydispersity, this value should be viewed as an estimate rather than a sharp threshold. In practice, pronounced interpenetration is observed only for ζ > 1, in agreement with previous simulations of realistic microgel models.^49^

The progressive reduction in particle size is accompanied by a marked increase in the structural organization. For small values of ζ, the system displays a disordered, fluid-like structure. As ζ increases, local ordering gradually emerges and becomes more pronounced, signaling a transition toward increasingly structured phases. While the size reduction is modest at low pressures, it becomes progressively more significant with increasing compression. This trend is quantified in Figure 9, which presents the particle size distributions for nominal volume fractions ranging from ζ = 0.49 to 9.52. Over this range, the average particle radius decreases from *R* ≃ *R* ^sw^ to *R* ≃ 0.6 *R* ^sw^, basically approaching the fully collapsed state.

**Figure 9:**
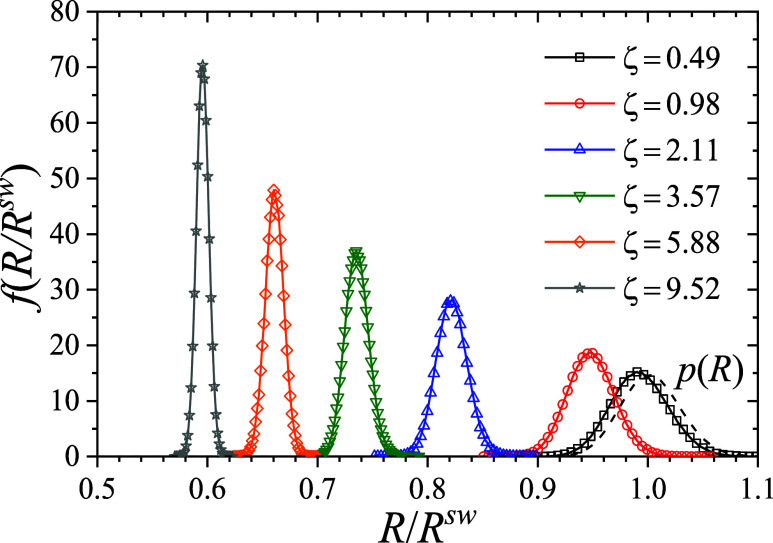
Lines with symbols show the probability size distribution *f*(*R*) from MC simulations of microgel suspensions at different nominal volume fractions, from ζ = 0.49 to 9.52. The dashed line shows the parent size distribution, *p*(*R*), obtained in the limit of an infinitely diluted microgel suspension. In all cases at T* = 0.9487 (i.e., *T* = 15 °C).

Alongside the reduction in the mean particle size, the size distribution exhibits a marked narrowing with increasing pressure. This effect is clearly visible in Figure 9, where higher volume fractions correspond to markedly less polydisperse populations. The resulting near-monodispersity at high compression not only facilitates more efficient packing but also enhances interparticle correlations, as evidenced by the emergence of well-defined peaks in the radial distribution functions shown in Figure 10.

**Figure 10:**
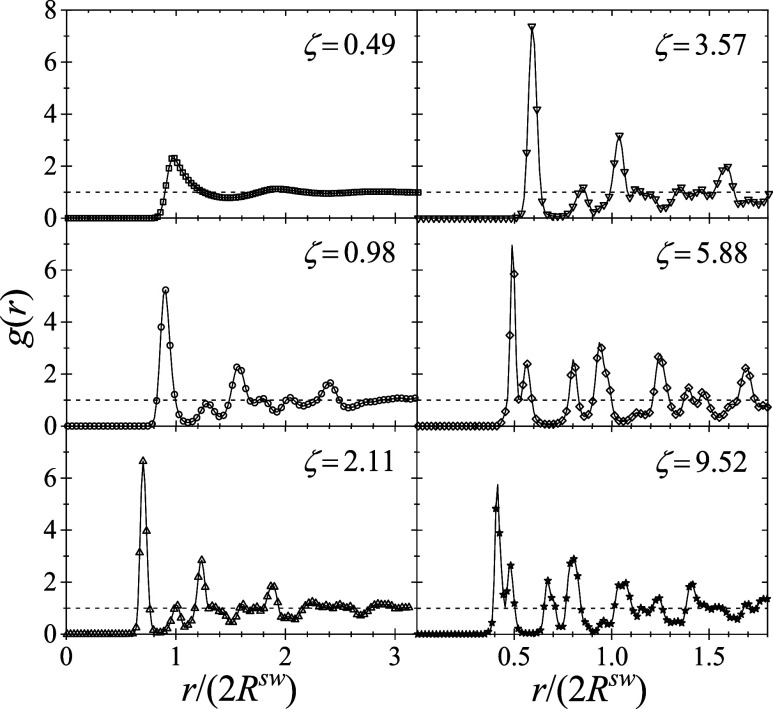
Radial distribution functions calculated from MC simulations of microgel suspensions at different nominal volume fractions, from ζ = 0.49 (top left) to 9.52 (bottom right). The horizontal dashed line indicates that *g*(*r*) = 1. In all cases at T* = 0.9487 (i.e., *T* = 15 °C).

As the concentration increases, the first peak of *g*(*r*) shifts to lower values of *r*/(2*R* ^sw^), reflecting the smaller particle size and the resulting closer proximity of neighboring particles. More importantly, particle correlations become stronger, as demonstrated by the emergence of increasingly sharp and well-defined peaks in *g*(*r*). This growing positional order with an increasing concentration signals the formation of more structured and densely packed arrangements. Examining the evolution of the peak structure in *g*(*r*) offers further insight into the system’s ordering. At the lowest volume fraction, ζ = 0.49 (upper left panel of Figure 10), *g*(*r*) displays the hallmark of a disordered fluid: a single prominent peak corresponding to the first neighbor shell, followed by a largely featureless profile indicative of weak long-range correlations. As ζ increases, additional peaks emerge, revealing the onset of long-range correlations typical of crystalline order.

Notably, the pattern and spacing of these peaks change nonmonotonically with increasing ζ. At intermediate volume fractions, between ζ = 0.98 and 3.57, the sequence and relative positions of peaks, partly influenced by continued particle shrinkage, differ markedly from those observed at higher volume fractions (ζ = 5.88 and 9.52). This shift suggests a transition between distinct crystalline structures, as the system becomes more densely packed. To confirm this transition and identify the stable structures in each case, we employed the polyhedral template matching (PTM) method,^87^ integrated into the OVITO software.^88^ This technique characterizes local crystal structures by comparing particle positions to reference templates of various crystalline phases, yielding the fraction of particles whose environments match each structure.

The PTM analysis corroborates the disordered nature of the fluid at low concentrations, consistent with the conclusions drawn from the radial distribution functions. However, the structural landscape evolves significantly as the pressure increases. At intermediate volume fractions, the system exhibits a coexistence of hexagonal close-packed (HCP) and face-centered-cubic (FCC) crystalline domains, each with varying defect concentrations. For example, at ζ = 3.57, approximately 47% of particles are identified as having an HCP local environment, while about 41% correspond to FCC-like surroundings. Particles exhibiting a body-centered cubic (BCC) structure account for less than 1% of the crystalline order, while the rest show no identifiable crystalline order. In contrast, at higher volume fractions (ζ = 5.88 and 9.52), the system undergoes a marked transition, with over 80% of particles adopting a BCC environment. The rest are predominantly disordered, with only a negligible fraction displaying HCP or FCC characteristics. These results were obtained using an RMSD cutoff of 0.1, following the guidelines recommended by Larsen and co-workers.^87^

The sequence of structural changes revealed by PTM analysis is consistent with trends observed in both experimental and numerical studies of microgel suspensions. Gasser et al.^89^ observed that the particle size of thermoresponsive PNIPAM microgels remains nearly constant up to volume fractions roughly between random close packing and space filling, and that shrinkage occurs at higher ζ with minimal interpenetration, highlighting the importance of particle softness in determining suspension behavior. Conley et al.^44,90^ later showed experimentally that under increasing compression, microgel particles first interpenetrate without significant shape deformation. Above ζ ∼ 1.3, particles begin to deform steadily until a maximum deformation is reached around ζ ∼ 1.75. Beyond this point, further densification occurs primarily through isotropic compression, with particle volume remaining nearly constant during deformation and a continuous reduction of particle size only at very high packing fractions (ζ ≥ 3). These findings are consistent with the more recent numerical analysis by Del Monte and Zaccarelli,^49^ which shows that isotropic shrinking occurs immediately above particle contact, followed almost simultaneously by deformation and interpenetration, with deformation saturating at very large ζ and further densification dominated by microgel shrinking and particle overlaps. Additionally, the crystalline phases predicted by simulations were confirmed in experiments. For instance, Marcelo et al.^91^ recently observed predominantly BCC structures, with a minor fraction of FCC, in concentrated PNIPAM solutions. Other studies^92^ have reported the stabilization of FCC/HCP coexistence under similar conditions. In contrast, Gasser et al.,^93^ investigating a different type of microgel, found HCP structures using neutron diffraction techniques. Overall, these experimental and numerical observations qualitatively support the sequence of transitions we observe in our simulations, emphasizing the predictive capability of our model, while also indicating that extreme overpacking may involve additional mechanisms not fully captured by our current approximations. However, they also underscore the open questions concerning the stability of the various crystalline phases, which depend on particle size and structure as well as the possible transitions between these phases.

## Conclusions

We have introduced a theoretical and computational framework tailored to describe core–shell microgels, whose internal architecture and compressibility are essential to fully understand their collective behavior. The formulation distinguishes between core and shell contributions to the intrinsic free energy, enabling a differentiated treatment of the mechanical response across regions of varying polymer density and cross-linking. This model allows us to determine how the core and shell sizes vary under thermal or mechanical compression by imposing an internal mechanical equilibrium within the microgel. We found that for small compressions, the outer radius *R* decreases while the core radius *R*_c_ remains nearly constant, highlighting the higher stiffness of the core relative to the shell. In contrast, at higher applied pressures, the system transitions into a distinct mechanical regime in which the core also experiences a pronounced decrease in size.

To model interactions in concentrated environments, we proposed a responsive multi-Hertzian pair potential that adapts to changes in the particle size. This approach goes beyond conventional models by incorporating both softness and compressibility in a size-dependent and thermodynamically consistent way. The model parameters were calibrated using experimental swelling data of PNIPAM microgels in dilute conditions, yielding plausible estimates of the solvency parameter χ across temperature.

Simulations based on the developed model reproduce key features observed in compressed microgel suspensions, such as the narrowing and shifting of the size distribution, and a nontrivial evolution of the effective packing fraction that agrees with previous simulation studies.^49^ We also find the emergence of a size–stiffness coupling that modulates structural correlations. These results confirm the critical role of particle-level responsiveness in dictating suspension properties beyond what nonresponsive fixed-shape or single-modulus models can capture.

Our model reveals rich phase behavior in the microgel fluid, marked by a transition between crystalline solids exhibiting distinct structural orders. This transition appears to be driven by two competing effects of compression: an increased packing density and reduced polydispersity. How exactly these factors interplay to induce the phase change remains an open question for future investigation. Our simulation results and PTM analysis align well with experimental studies showing that, upon compression, microgels transition from interpenetration to deformation and ultimately isotropic shrinking at high packing fractions, with particle softness governing suspension behavior.^44,89,90^ Notably, some of the identified phases resemble those reported for Hertzian sphere fluids,^94^ despite the latter’s nonresponsive particle nature. The presence of mixed FCC and HCP structures at intermediate pressures is also intriguing. Given the close similarity of these two crystal lattices, which exhibit minimal free energy differences in well-studied systems such as Lennard–Jones^95^ and hard spheres,^96^ it is unclear whether this reflects true phase coexistence or a transient, possibly metastable state. The complex phenomenology uncovered highlights both the fundamental interest in microgel suspensions and the need for further research to fully elucidate their phase behavior.

Our findings highlight the importance of incorporating internal heterogeneity and thermodynamic responsiveness in coarse-grained descriptions based on effective pair potentials to describe soft colloidal systems. The model presented here provides a versatile foundation for studying microgels under confinement, crowding, or external stimuli and can be extended to address out-of-equilibrium or multicomponent situations in future work.

We acknowledge that our analytical treatment relies on the assumption of a constant polymer density and a uniform distribution of cross-linkers within the shell, whereas experimental PNIPAM microgels typically exhibit a gradual decrease of polymer density with increasing distance from the core.^63^ While a more refined description accounting for this nonuniformity would lead to a more complex, likely nonanalytical, form of the effective microgel–microgel interaction potential, the comparison with experimental data indicates that such additional complexity is not required for the systems studied here. In fact, previous simulations have shown that the effective interactions can be accurately captured by analytical multi-Hertzian potentials with a few additive terms, which successfully reproduce structural observables such as radial distribution functions (see refs 49,66 ). An additional limitation of our model is that it considers particles to be spherical at all times, when various studies have shown that deformation can be significant at high packing densities^32^ The study of how this change in shape influences the behavior of the fluid is beyond the scope of our work.

As a perspective for future work, our theoretical model based on responsive effective pair potentials could be extended to study microgels adsorbed at fluid–fluid or fluid–air interfaces. In these systems, recent studies have shown that the Hertzian or multi-Hertzian description often provides a good representation of particle interactions, due to the enhanced stiffness of the polymer network at the interface.^97,98^ Therefore, applying our framework to interfacial microgels represents a promising direction for future investigations, potentially connecting the detailed internal microgel structure to collective interfacial properties.

Another possible extension of the present work would be the application of our theoretical framework to copolymer microgels.^99^ In principle, the Flory–Rehner model can be generalized to account for the presence of multiple monomer species by introducing an effective polymer–solvent interaction parameter, χ_eff_, that combines the contributions of the individual monomers and their mutual interactions. For random copolymer microgels with a homogeneous distribution of monomers, this generalization is straightforward and would only require replacing χ by χ_eff_ in the free-energy expressions. However, for systems with strong compositional heterogeneities, such as block copolymer microgels or microphase-separated architectures, a more detailed description would be needed, including spatial variations of the polymer density and possibly interfacial contributions.
